# Dynamic multi-segmental postural control in patients with chronic non-specific low back pain compared to pain-free controls: A cross-sectional study

**DOI:** 10.1371/journal.pone.0194512

**Published:** 2018-04-10

**Authors:** Michael A. McCaskey, Brigitte Wirth, Corina Schuster-Amft, Eling D. de Bruin

**Affiliations:** 1 Department of Health Sciences and Technology, Institute for Human Movement Sciences, ETH Zurich, Zurich, Switzerland; 2 Research Department, Reha Rheinfelden, Rheinfelden, Switzerland; 3 Institute of Rehabilitation and Performance Technology, Bern University of Applied Sciences, Burgdorf, Switzerland; 4 Department of Chiropractic Medicine, University of Zurich, Balgrist University Hospital, Zurich, Switzerland; 5 Department of Neurobiology, Care Sciences and Society, Division of Physiotherapy, Karolinska Institutet, Huddinge, Sweden; University of Memphis, UNITED STATES

## Abstract

Reduced postural control is thought to contribute to the development and persistence of chronic non-specific low back pain (CNLBP). It is therefore frequently assessed in affected patients and commonly reported as the average amount of postural sway while standing upright under a variety of sensory conditions. These averaged linear outcomes, such as mean centre of pressure (CP) displacement or mean CP surface areas, may not reflect the true postural status. Adding nonlinear outcomes and multi-segmental kinematic analysis has been reported to better reflect the complexity of postural control and may detect subtler postural differences. In this cross-sectional study, a combination of linear and nonlinear postural parameters were assessed in patients with CNLBP (n = 24, 24-75 years, 9 females) and compared to symptom-free controls (CG, n = 34, 22-67 years, 11 females). Primary outcome was postural control measured by variance of joint configurations (uncontrolled manifold index, UI), confidence ellipse surface areas (CEA) and approximate entropy (ApEn) of CP dispersion during the response phase of a perturbed postural control task on a swaying platform. Secondary outcomes were segment excursions and clinical outcome correlates for pain and function. Non-parametric tests for group comparison with P-adjustment for multiple comparisons were conducted. Principal component analysis was applied to identify patterns of segmental contribution in both groups. CNLBP and CG performed similarly with respect to the primary outcomes. Comparison of joint kinematics revealed significant differences of hip (*P* < .001) and neck (*P* < .025) angular excursion, representing medium to large group effects (*r*′*s* = .36 − .51). Significant (*P*′*s* < .05), but moderate correlations of ApEn (r = -.42) and UI (r = -.46) with the health-related outcomes were observed. These findings lend further support to the notion that averaged linear outcomes do not suffice to describe subtle postural differences in CNLBP patients with low to moderate pain status.

## Introduction

Chronic non-specific low back pain (CNLBP) is believed to develop in about 10% of people who experience some form of acute low back pain in their life-time [1]. As acute low back pain occurs in almost 84% of the population [2], CNLBP is a highly prevalent symptom causing troubling global socio-economic burdens through direct or indirect costs [2, 3]. Recent systematic reviews have estimated the mean point prevalence of CNLBP at 18.3% to 23% [3, 4], although there seem to be large variations related to economic status, gender, and age [1]. For instance, while younger individuals under 29 are less commonly affected (4.2% to 10.2%), prevalence has been reported to be up to four times higher in people aged 60 and older [1]. Despite recent advances towards the understanding of the underlying mechanism, CNLBP remains a disabling condition limiting daily activities of affected people [5]. Since 1990, the reported disability-adjusted life years have increased by approximately 42%, positioning it at the highest ranked cause of years lived with disability in the Global Burden of Disease 2010 Study [4]. Accordingly, evaluating possible causes and associated mechanisms of CNLBP has been, and remains, a priority in the field of musculoskeletal research [3]. As CNLBP cannot be attributed to a recognizable, specific pathology [2], researchers have turned their attention to psychosocial factors, such as fear avoidance, central sensitization and resulting changes in movement behaviour [6]. Particularly aberrant postural control observed in patients with CNLBP has been suggested to be a possible factor in its aetiology [7]. Postural control is a common outcome reported in assessments to quantify functional instability associated with pain or to prescribe appropriate treatments [7, 8]. However, there have been highly inconsistent findings regarding its validity [7, 8].

Postural control involves complex regulatory feedback systems which rely on continuous and non-corrupted signalling of afferent information [9, 10]. Lack of dynamic and variable sensorimotor input has been described as a possible origin of CNLBP, as it could impair sensorimotor accuracy needed to adopt the correct posture in a variable environment [11, 12]. From neurophysiological findings, it is known that trunk muscle activation patterns change with low back pain (LBP), leading to altered postural responses with potentially pain exasperating consequences [13]. Addressing the issue of causality, a series of studies have shown reduced adaptability of postural control strategies in young LBP patients. In a longitudinal 2-year follow-up study, it was found that symptom-free participants with postural strategies similar to LBP patients were at greater risk to develop CNLBP [14]. Recently identified reorganizations of specific sensorimotor areas associated with the performance of a dynamic postural control task [15] lend further support to earlier theories by Janda [11], who claims that people with coordination difficulties are more likely to develop pain.

Postural control is defined as the ability to coordinate all segments of the body to maintain control of the body’s centre of mass (CM) in relation to the base of support [10]. A common way to assess postural control is measuring the amount of CM sway indirectly. The trajectory of the centre of pressure (CP) is strongly associated with the CM and can be recorded from force- or pressure plates mounted onto the base of support [16]. One of many widely-accepted metrics to report the amount of sway is the area of the 95% confidence ellipse (CEA) fitted to the bidimensional plane of the CP projection [17]. The prevailing hypothesis is, that an increased CEA represents poorer postural control. Whereas an overwhelming majority of findings suggest changes in postural control are associated with LBP [8, 13], it remains disputable whether the use of linear outcomes alone, such as CEA, can capture the complexity of this motor task [7]. Reducing postural reactions to single outcomes may not reflect postural strategies, which vary greatly between individuals [18]. As has been pointed out by Mazaheri et al. [7], the assessment of postural control should, therefore, be complemented by dynamic nonlinear measures of posture. Linear measures merely represent the magnitude of CP variation and assume that the variance of a time-series is random error. Approximate Entropy (ApEn) is a nonlinear measure that reflects this variance, suggesting movement variability may also be purposeful to accurately and efficiently perform dynamic movements [19]. Findings from clinical studies lend further support to this idea, where CP variability in athletes with concussion deviated from values observed in healthy participants [20, 21].

One limitation of CP based measures is that they summarise the contribution of all body segments as the global ground reaction force recorded by the sensor plate [16]. While this would be informative under the assumption of the inverted pendulum model, where postural control is primarily stabilized at the ankle or hip joint [22], it does not suffice to describe the origin of postural deficiencies in a multidimensional postural model [23]. Recent findings, however, suggest postural control is a multi-segmental task involving most weight-bearing joints of the human body [23–25]. This has led to a number of studies investigating multi-joint coordination patterns using kinematic synergies to deal with the redundancy problem and account for its functional advantage, i.e. adaptive flexibility through redundancy [26–28]. One such method is the uncontrolled manifold analysis (UCM), which allows linking of multi-dimensional elemental variables to a one-dimensional performance variable [24]. UCM is based on the idea that the central nervous system (CNS) does not control the exact movement of every peripheral joint segment. Instead, it merely tries to limit undesirable variation in segmental configuration which would impair the accuracy of the desired goal (nonmotor equivalent). In terms of postural control, the goal would be to maintain the CM within the area of base of support by limitation of all possible joint configurations deviating from this goal [29]. Providing a manifold of solutions that agree with the endpoint (motor equivalent variability), the UCM approach offers a solution to the problem of inverse kinematics where an under-defined system with more than one solution must be analysed [24].

It is not until only recently that multi-segmental analysis of postural control with UCM has been applied to pathological conditions [30, 31]. For instance, in children with Down-Syndrome, the ratio of motor equivalent and nonmotor equivalent variability is lower when walking on a treadmill as compared to healthy controls [30]. In an analysis of a sit-to-stand task, Tajali et al. found significantly lower motor equivalence in CNLBP [31]. But it is upright standing where the importance of control of CM for postural stability is best documented in healthy populations [27, 32, 33], yet no comparisons have investigated how chronic pain may affect strategies underlying the control of the CM while standing upright. Thus, there is demand for outcomes that include information from segmental variation and signal structure, where more subtle differences are expected to be detectable [7, 34, 35]. The primary goal of the present study was, therefore, to use a combination of linear and nonlinear CP-based measures and UCM analysis as an indicator for postural control deficiencies in patients with CNLBP. It was hypothesised that patients with CNLBP would perform poorer in a postural control task when compared to symptom-free controls (CG). This would be reflected by proportionally more nonmotor equivalent segmental variability representing the structural pattern of the postural response, i.e. a lower relative ratio of variance components (UI), a significantly lower (too rigid) or higher (too chaotic) ApEn and larger CEA.

## Materials and methods

**Fig 1:**
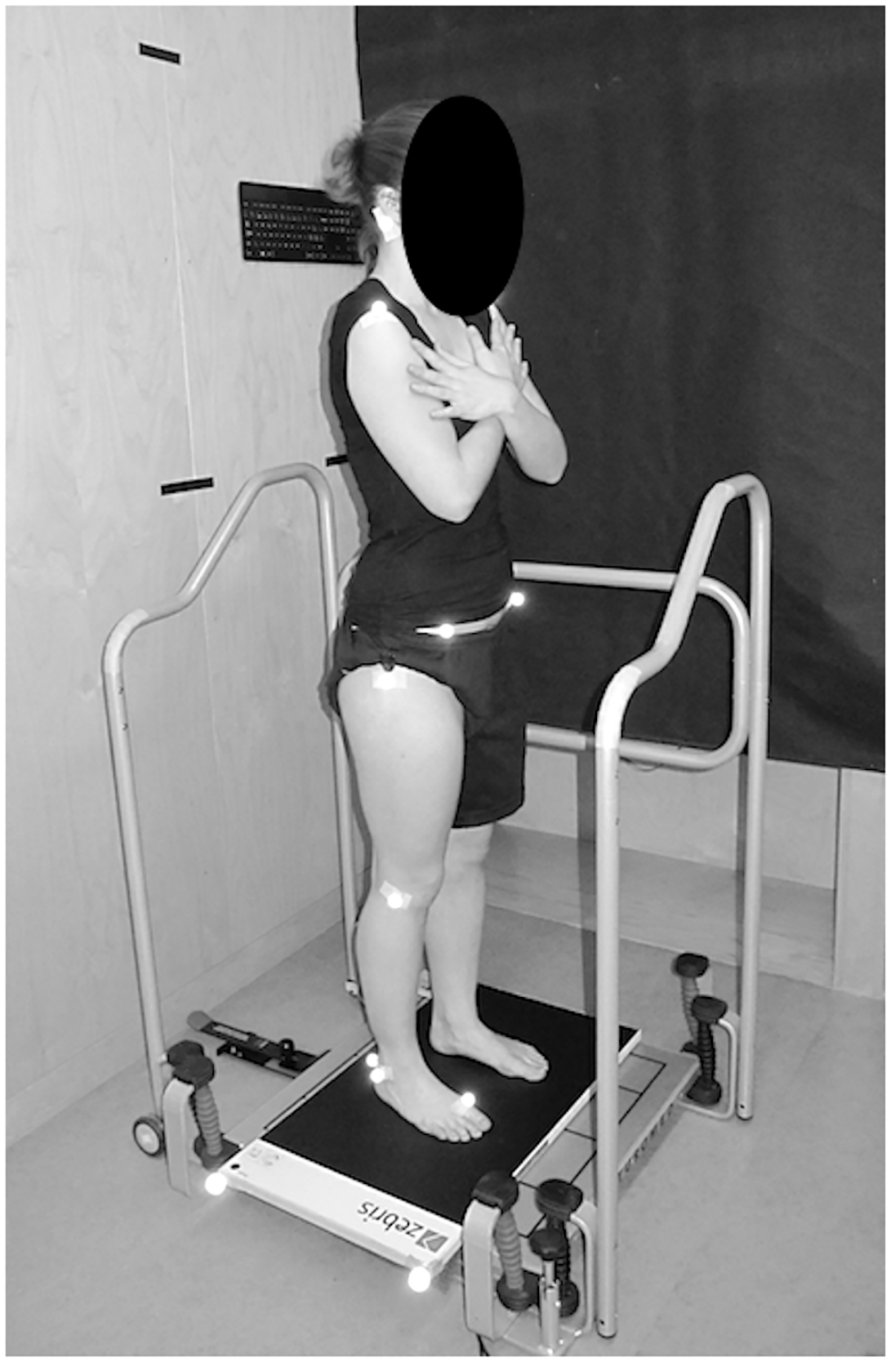
Illustration of the measurement setup.

The procedures of this cross-sectional study have been approved as part of a larger randomized controlled trial by the local ethics committee (EC North-Western Switzerland, EC number: 2014-337). The mentioned randomized controlled trial has been registered (ClinicalTrials.gov, NCT02304120) and its protocol published [36]. The current article presents and discusses baseline comparison of included patients and healthy participants for primary and secondary parameters, but does not cover longitudinal data or proprioceptive comparison of cervical repositioning error mentioned in the trial protocol. The latter findings are currently being prepared for submission and shall be presented elsewhere. The study conforms to the guidelines of Good Clinical Practice E6 (R1) and the Declaration of Helsinki (2013). No data was recorded before written informed consent was given by the participants. The individual depicted in Fig 1 in this manuscript has given written informed consent (as outlined in PLOS consent form) to publish these case details.

### Study population

Upon public announcement, adult pain-free controls and patients (≥ 18 years) with confirmed symptoms of CNLBP presented for assessment at the study site, a rehabilitation centre in Switzerland. Included patients reported enduring pain symptoms localized primarily below the costal margin and above the inferior gluteal folds for more than 3 months [2, 37]. Patients were excluded if they presented with nerve root pain or specific spinal pathology (e.g. infection, tumour, fracture, scoliosis). Further exclusion criteria were: history of spinal surgery (e.g. decompensation); whiplash incidence within the last 12 months; known vestibular or other neurological pathologies; inability to follow the procedures of the task. Participants of the CG confirmed to be pain-free with no limitation in all areas of daily activity. Age, weight, and pain levels were recorded for all participants (see Table 1).

**Table 1:** Mean and range values for characteristics of the study population.

	Units	Symptom-free group (N = 34, 9 female)	CNLBP group (N = 24, 11 female)
Age (range)	years	39.5 (22-67)	53.2 (24-75)
Height (SD)	cm	171.2 (9.2)	171.6 (10.0)
Weight (SD)	kg	68.3 (11.0)	71.4 (11.2)
VAS (SD)	%	0.0 (0)	28.9 (22.2)
ODI (SD)	%	0.0 (0)	20.1 (10.1)

CNLBP: Chronic non-specific low back pain; SD: Standard deviation; VAS: Visual Analogue Scale for self-reported pain; ODI: German version of the Oswestry Disability Questionnaire.

### Procedures

Postural control was assessed on a labile platform fixated at 3cm deflection in posterior direction (Posturomed™, Haider Bioswing GmbH, Pullenreuth, Germany). Upon manual release, the platform sways predominately in anteroposterior direction restricted to the horizontal plane. All the device’s damping brakes were released to allow maximal sway and provoke sufficient postural response. Participants were instructed to adopt an upright posture with arms folded across the chest, feet pointed in a natural stance and gaze fixed on a black dot straight ahead. On the cue ‘ready-steady-go’, the assessor released the platform. Subjects were asked to react naturally to this anticipated perturbation, as they would do when standing in a vehicle coming to a slow stop. Two familiarisation trials were performed prior to the five measurement trials. Participants could relax in-between trials and lean on the security bars of the device. But they were also instructed not to move away from the initial foot position. Fig 1 illustrates the setup for the postural control task.

### Study equipment

**Fig 2:**
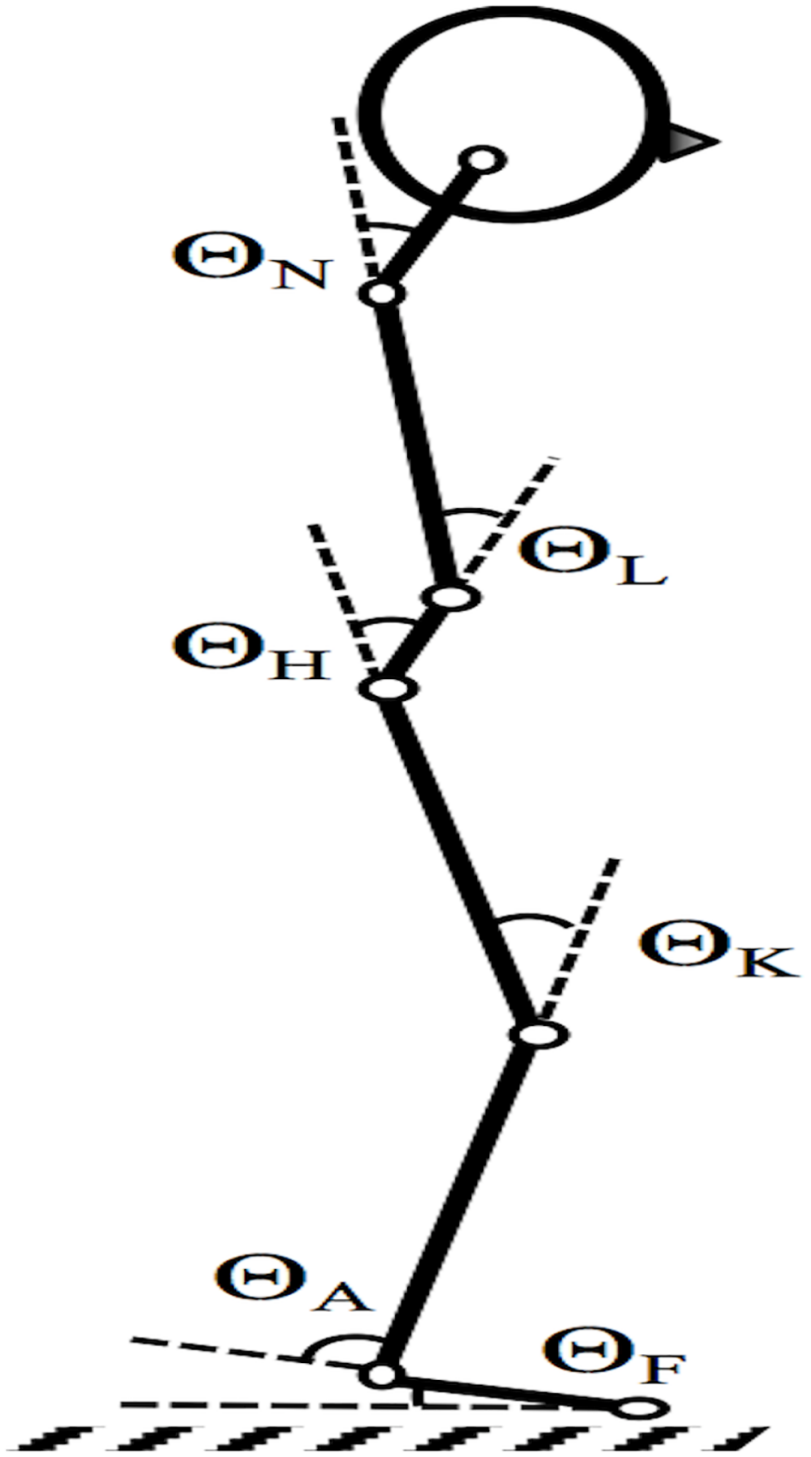
Schematic representation of the defined segment angles. Θ_*N*_ = Neck angle; Θ_*L*_ = Lumbar angle; Θ_*H*_ = Hip angle; Θ_*K*_ = Knee angle; Θ_*A*_ = Ankle angle; Θ_*F*_ = Foot angle; Marker positions (from head to toe): corner of the eye (orbital process of the zygomatic bone), acromion, anterior superior iliac spine, greater trochanter, lateral condyle of femur, lateral malleolus, 1st metatarsal bone.

Two-dimensional marker trajectories in space were collected at a sampling frequency of 100Hz by two cameras for frontal and sagittal view (1200x720 spatial resolution) [38, 39]. Motion data were recorded with Templo v.8.2 (Contemplas GmbH, Kempten, Germany). Seven sagittal retroreflective markers were applied (see Fig 2): mastoid process, acromion, hip (greater trochanter and anterior superior iliac spine), knee, ankle, and toe. Finally, CP was recorded using the Zebris FDM-S pressure plate (sampling frequency 60 Hz, Zebris Medical GmbH, Isny im Allgäu, Germany), which was placed on top of the swaying platform. All final analysis algorithms were implemented and executed in Matlab™ version R2017a (Mathworks Inc., Natick, MA, USA).

### Data processing

**Fig 3:**
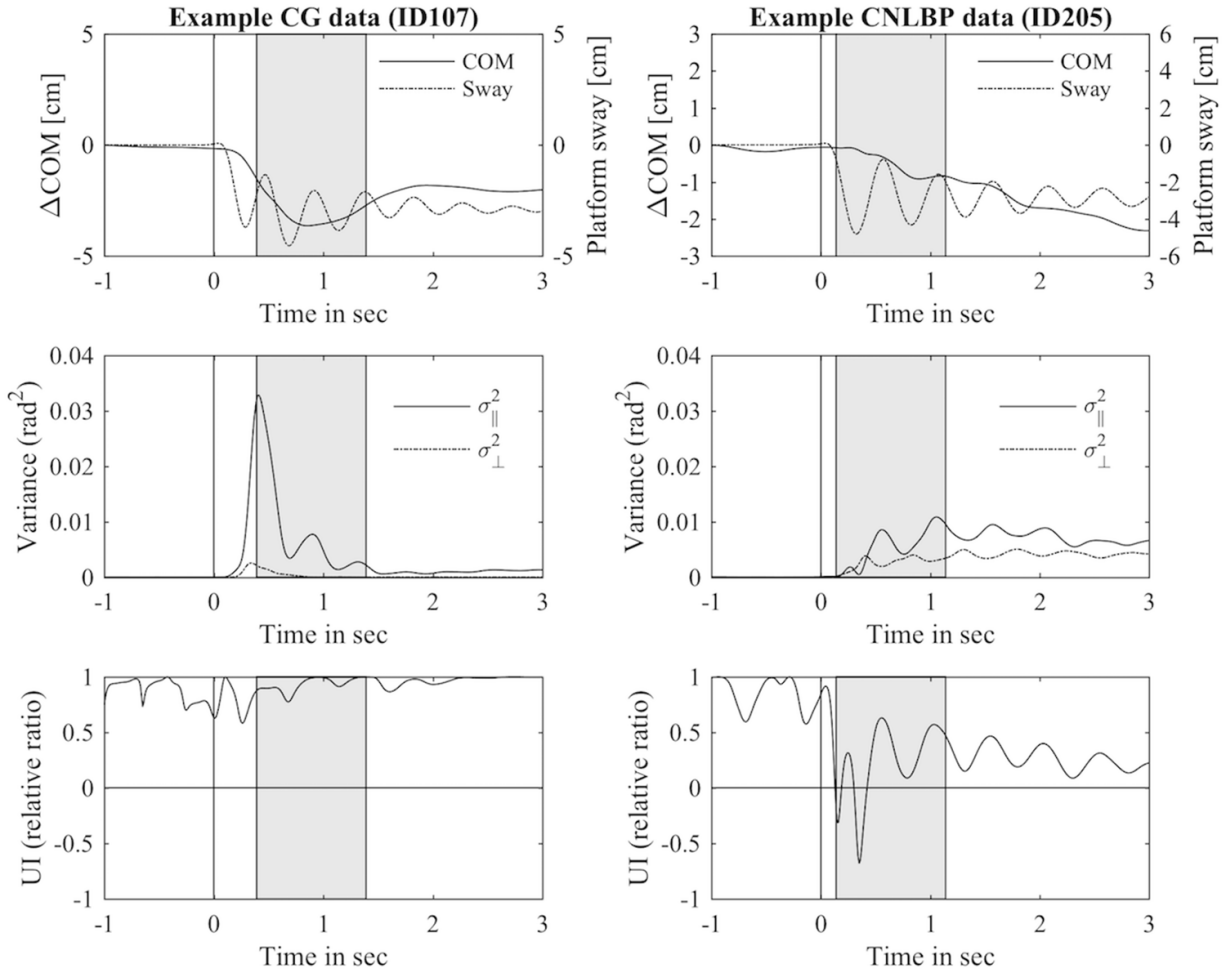
Example data for kinematic analysis. Data of a medium performer (mean *UI* = .55) from the symptom-free CG (left) and low performer (mean *UI* = −.47) from the CNLBP group (right). The solid vertical lines indicate time point of platform release. The shaded areas indicate the active response phase (area of interest). The top panel shows CM trajectory and actual platform sway trajectory. The middle panel shows the normalized variance within and perpendicular to pre-perturbation joint configuration space. Lower panel shows the relative ratio of variance.

The recording of kinetic and kinematic data started simultaneously, shortly before the assessor released the platform, and stopped automatically after ten seconds. To synchronise both recordings, the data was later time-normalised and aligned along the moment of perturbation. The recordings were then trimmed to one second pre-perturbation and three seconds post-perturbation. To account for the individual time needed to actively react to the mechanical perturbation as a corrective response [40], an active response phase was derived from the kinematic data [41]. The beginning of the active response phase was defined as first zero-crossing of the CM acceleration after perturbation and ended one second later (see Fig 3). The dependent variables (described below) were calculated during this active response phase only. Coordinate data of each reflective marker were filtered at 5 Hz using a bi-directional, second-order, Butterworth digital filter [32]. For calibration purposes, fixed geometrical objects with known metrics and fixed angles were placed onto the labile platform and recorded from both perspectives.

#### Joint angles and centre of mass excursion

As shown in Fig 2, the sagittal marker coordinates were used to calculate the joint angles of the foot (*θ*_*F*_), ankle (*θ*_*A*_), knee (*θ*_*K*_), hip (*θ*_*H*_), lumbar (*θ*_*L*_) and neck (*θ*_*N*_) [10]. Based on estimated segmental CM and mass proportions, weighted sagittal plane CM location was computed for every frame [10]. A geometrical model relating the CM to the joint configuration with origin at the toe was expressed through a trigonometric analysis (Eq 1):

$$\begin{array}{c} \begin{array}{cc} CM_{x}= & m_{1}\left(d_{1}l_{1}cos\left(\theta_{F}\right)\right)+\\ & m_{2}\left(l_{1}cos\left(\theta_{F}\right)+d_{2}l_{2}cos\left(\theta_{F}+\theta_{A}\right)\right)+\\ & m_{3}\left(l_{1}cos\left(\theta_{F}\right)+l_{2}cos\left(\theta_{F}+\theta_{A}\right)+d_{3}l_{3}cos\left(\theta_{F}+\theta_{A}+\theta_{K}\right)\right)+\\ & m_{4}\left(l_{1}cos\left(\theta_{F}\right)+l_{2}cos\left(\theta_{F}+\theta_{A}\right)+l_{3}cos\left(\theta_{F}+\theta_{A}+\theta_{K}\right)\right)+\\ & \;d_{4}l_{4}cos\left(\theta_{F}+\theta_{A}+\theta_{K}+\theta_{H}\right)+\\ & m_{5}\left(l_{1}cos\left(\theta_{F}\right)+l_{2}cos\left(\theta_{F}+\theta_{A}\right)+l_{3}cos\left(\theta_{F}+\theta_{A}+\theta_{K}\right)\right)+\\ & \;l_{4}cos\left(\theta_{F}+\theta_{A}+\theta_{K}+\theta_{H}\right)+d_{5}l_{5}cos\left(\theta_{F}+\theta_{A}+\theta_{K}+\theta_{H}+\theta_{L}\right)+\\ & m_{6}(l_{1}cos\left(\theta_{F}\right)+l_{2}cos\left(\theta_{F}+\theta_{A}\right)+l_{3}cos\left(\theta_{F}+\theta_{A}+\theta_{K}\right)+\\ & \;l_{4}cos\left(\theta_{F}+\theta_{A}+\theta_{K}+\theta_{H}\right)+l_{5}cos\left(\theta_{F}+\theta_{A}+\theta_{K}+\theta_{H}+\theta_{L}\right)+\\ & \;d_{6}l_{6}cos\left(\theta_{F}+\theta_{A}+\theta_{K}+\theta_{H}+\theta_{L}+\theta_{N}\right)) \end{array} \end{array}$$ (1)

where *m*_*i*_ is the *i*^*th*^ segment proportional mass expressed as percentage of total body mass, *l*_*i*_ is the *i*^*th*^ segment’s length, *d*_*i*_ is the distal distance from the CM of the *i*^*th*^ segment expressed as a percentage of its length, where *i* = (1, …, 6) = (*foot*, *shank*, *thigh*, *pelvis*, *trunk*, *neck*). The joint angles were primarily used to examine the relation of the elemental variables *θ*_*i*_ with the performance variable *CM*_*x*_. Displacement of *CM*_*x*_ and joint angle excursion were calculated as the approximate integral of their trajectories.

#### Components of joint angle variability

For the present study, a variant of the UCM approach, proposed by Scholz et al. [32], was used. Here, the measure of multi-segmental CM control is evaluated at each instant in time to analyse postural responses in different phases during the postural task. For every recorded frame the variance of the control variables (i.e. joint angles) across the attempts can be partitioned into two components: parallel and orthogonal to the UCM (see below). The variance of the performance variable CM orthogonal to the UCM is usually smaller as compared to the variance parallel to it when standing in response to surface perturbation [32]. Both components of joint angle variability were computed to quantify the amount of variability causing unwanted change (nonmotor equivalent) and the amount of variability returning the CM to its steady-state position (motor equivalent). The relative ratio of both components was reported to allow group-wise comparison. Exemplary data is presented in Fig 3. To obtain the variance of both components, the following steps were applied [32]:

1. Create geometric model (Eq 1).
2. Compute reference joint-configuration based on mean joint configuration during 1 second prior to perturbation across trials.
3. Compute the joint deviation vector (JDV) as the difference between the current joint-configuration and the reference joint-configuration for each segment ${\bar{\theta }}_{i}$ at every time-frame of the recording:
  $$\begin{array}{c} JDV=[\begin{array}{c} \theta_{F}-{\bar{\theta }}_{F}\\ \theta_{A}-{\bar{\theta }}_{A}\\ \theta_{K}-{\bar{\theta }}_{K}\\ \theta_{H}-{\bar{\theta }}_{H}\\ \theta_{L}-{\bar{\theta }}_{L}\\ \theta_{N}-{\bar{\theta }}_{N} \end{array}] \end{array}$$ (2)
4. Linearize the UCM to relate non-commensurate units with different numbers of degrees of freedom through the definition of the Jacobian matrix *J*(*θ*) and the computation of its null space around the reference configuration, *N*(*J*).
  $$\begin{array}{c} 0=J\left(\bar{\theta }\right)\epsilon_{n-d}=[\begin{array}{cccccc} \frac{\delta CM_{x}}{\delta \theta_{F}} & \frac{\delta CM_{x}}{\delta \theta_{A}} & \frac{\delta CM_{x}}{\delta \theta_{K}} & \frac{\delta CM_{x}}{\delta \theta_{H}} & \frac{\delta CM_{x}}{\delta \theta_{L}} & \frac{\delta CM_{x}}{\delta \theta_{N}} \end{array}]\epsilon_{n-d} \end{array}$$ (3)
  $$\begin{array}{c} N=[\begin{array}{ccccc} \epsilon_{1F} & \epsilon_{2F} & \epsilon_{3F} & \epsilon_{4F} & \epsilon_{5F}\\ \vdots & \vdots & \vdots & \vdots & \vdots \\ \epsilon_{1N} & \epsilon_{2N} & \epsilon_{3N} & \epsilon_{4N} & \epsilon_{5N} \end{array}] \end{array}$$ (4)
  where *ϵ*_*n*−*d*_ are the basis vectors of the null space (*n* is the number of elemental variables and *d* is the number of dimensions of the performance variable) representing the linear subspace of all joint-configurations that leave the *CM*_*x*_ position unchanged.
5. Decomposition of the JDV projection into the null-space (*θ*_||_ and into its orthogonal space *θ*_⊥_:
  $$\begin{array}{c} \theta_{\left|\right|}=\sum_{i=1}^{n-d}({N\left(J\right))}_{i}^{T}\cdot JDV)N{\left(J\right)}_{i} \end{array}$$ (5)
  $$\begin{array}{c} \theta_{⊥}=JDV-\theta_{\left|\right|} \end{array}$$ (6)
  The computed scalar values represent the length of projection to quantify the consistency of the instantaneous joint configuration with the steady-state configuration.
6. Calculate variance normalised to the number of degrees of freedom (*n* − *d*) and trial length (N):
  $$\begin{array}{c} \sigma_{\left|\right|}^{2}=\frac{\sum_{i=1}^{N}\theta_{||N}^{2}}{(n-d)N} \end{array}$$ (7)
  $$\begin{array}{c} \sigma_{⊥}^{2}=\frac{\sum_{i=1}^{N}\theta_{||N}^{2}}{dN} \end{array}$$ (8)
7. Calculate relative variance as UCM-index (UI) with values ranging from -1 to 1 [28]:
  $$\begin{array}{c} UI=\frac{\sigma_{\left|\right|}^{2}-\sigma_{⊥}^{2}}{\sigma_{\left|\right|}^{2}+\sigma_{⊥}^{2}} \end{array}$$ (9)

#### Pain and functional status

Self-reported impairment in daily activities was assessed using the German version of the Oswestry Disability Index (ODI-G) [42]. The ODI-G has shown to be a valid and reliable tool to assess functional status in a German-speaking study population [43]. The total score is reported in percentage of the total achievable 50 points (from 0% = minimal impairment to 100% = bedridden). Additionally, self-reported pain was recorded on a 100mm Visual Analogue Scale (VAS) with two endpoints representing the extreme states ‘no pain’ and ‘pain as bad as it could be’.

#### Centre of pressure

Several CP quantifying parameters have been suggested in the literature [7]. For the purpose of this study, CP 95% confidence-ellipse area (CEA) [17] was analysed as a measure of magnitude. Approximate entropy (ApEn) with dimensionality 2 and a tolerance of.2 times the standard deviation was analysed to quantify regularity of the time series, which has been reported to be more sensitive than magnitude alone [35]. Highly predictable time-series are reflected by a lower ApEn value suggesting rigid movement patterns. More chaotic and unpredictable data would be represented by a higher ApEn value, as would be expected from excessive and uncontrolled movement [18].

### Statistical analysis

Average values over five trials were used for kinematic and kinetic variables (UI, CEA, ApEn, and joint angle trajectories). Multivariate normality and homogeneity of variance was tested and had to be refuted. Hence, non-parametric comparison of two independent groups was computed using the Wilcoxon-Mann-Whitney statistics. To analyse the individual joint segments, principal component analysis (PCA) of the mean raw angles was calculated in order to identify the segments that contributed to overall variances in both groups. To reduce the number of dependent variables, only the joint angle trajectories of the principal components were compared between the groups. The principal components were computed from a data matrix of 60x6 for the active response phase, i.e., 60 participants and 6 angles. The percent of cumulated variability explained by each principal component was calculated for each time window. The overall mean PCA values are based on mean absolute PCA values of each participant and are presented per group. Significant contributions of segments to each principal component was indicated if its loading coefficient was greater than or equal to 0.5 [27, 33]. PCA was conducted on Matlab™ version R2017a (Mathworks Inc., Natick, MA, USA). Level of significance for the directional hypothesis testing was set to *α* = .05/2) and was adjusted for multiple comparisons of between-group differences using the Benjamin-Hochberg method [44] and the adjusted p-values are reported (*P*). Correlational effect sizes were calculated for each comparison (*r*). Spearman’s rho test was used to study the associations of functional outcomes and pain status with dependent variables within the CNLBP group. Due to significant age difference between groups, a sub-group analysis with homogeneous age comparison was computed to confirm findings. Averaged values for both groups were statistically analysed using R 3.3.2 running on RStudio (version 1.0.136, 2016, RStudio Inc., Boston).

## Results

### Postural control

**Fig 4:**
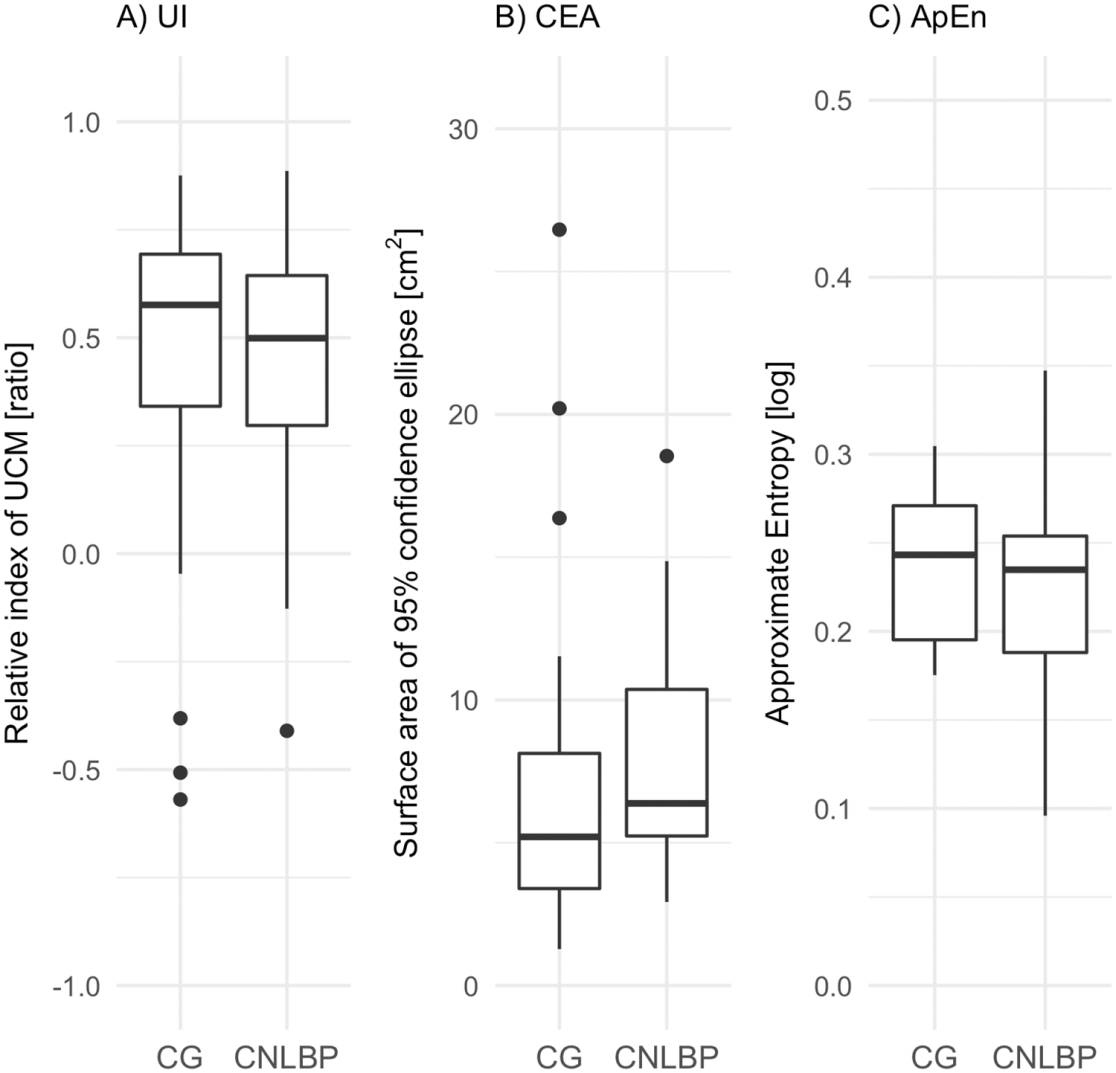
Group comparison of postural outcome measures. Across trials mean values of primary outcomes during active response phase after platform release. CG = control group; CNLBP = Chronic non-specific low back pain group; UI = Uncontrolled Manifold Index; CEA = 95% confidence ellipse surface area; ApEn = approximate entropy of antero-posterior centre of pressure signal.

**Fig 5:**
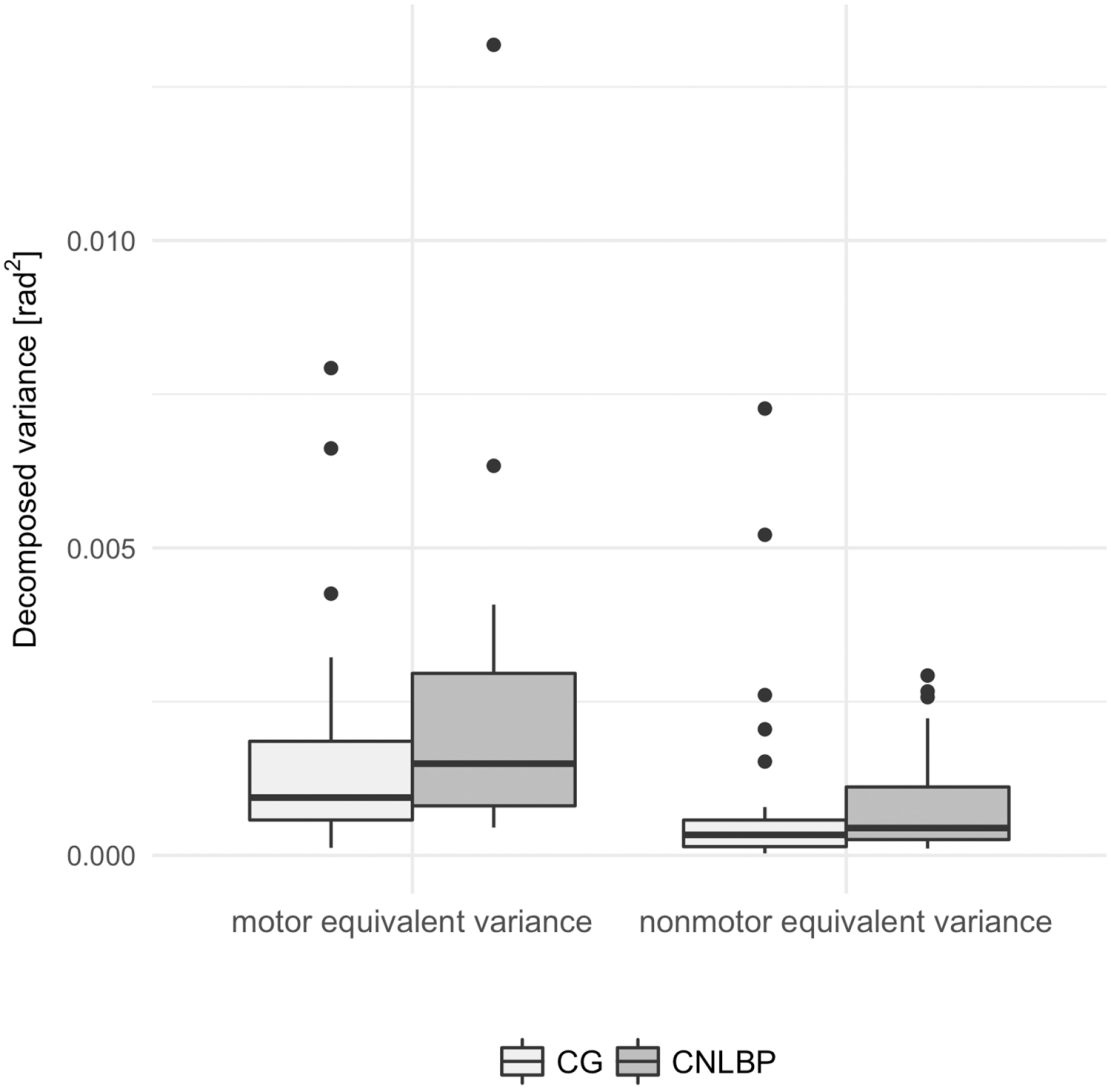
Group comparison of variance components. Across trials mean variance components during the first second of the active response phase after platform release. CG = control group; CNLBP = Chronic non-specific low back pain group.

On average, both groups performed with a UI greater than 0, which suggests the use of more motor equivalent joint configurations during the task in both groups than nonmotor equivalent configurations. Although patients with CNLBP had a slightly lower UI (*Mdn* = .47) when compared to CG (*Mdn* = .51), this difference was not significant, *W* = 441, *z* = −1.03, .37, *r* = .10 (see Fig 4A). When decomposing the UI into its variance components, a notably higher variance of nonmotor equivalence ($\sigma_{⊥}^{2}$) was observed in the CNLBP group (*Mdn* = 4.45x10^−4^) than in the pain-free CG (*Mdn* = 3.3x10^−4^), *W* = 302, *P* = .05 with medium-sized effect of *r* = .26 (see Fig 5). The variance within motor equivalence ($\sigma_{\left|\right|}^{2}$) was similar in both groups (*Mdn* = 1.49x10^−3^*vsMdn* = .94x10^−3^, *W* = 294, *P* = .97).

Similarly, the analysis of the CP data resulted in no significant difference. Regarding the measure of magnitude, there was a tendency towards a greater CEA in the CNLBP group (*Mdn* = 7.70*cm*^2^) than in the CG (*Mdn* = 5.75*cm*^2^), *W* = 280, *z* = −2.29, *P* = .04, *r* = .30 (see Fig 4B). As a measure of the structure, predictability and regularity (ApEn) of the antero-posterior CP signal was not different in the CNLBP group (*Mdn* = .23) compared to CG (*Mdn* = .24) during the active response phase, *W* = 451, *z* = −.1.14, .37, *r* = .15 (see Fig 4C).

Due to the significant age difference between the groups, an exploratory sub-group analysis of the main outcomes was conducted with all participants older than 30 years (CG: n = 21; mean age ±*SD* = 48 ± 13; CNLBP: n = 23; mean age±*SD* = 55 ± 14; t = -1.60, *ns*). The parallel comparison did not result in any difference regarding the results of the primary outcomes (adjusted *Ps* > .05).

### Segmental joint angle excursions

**Fig 6:**
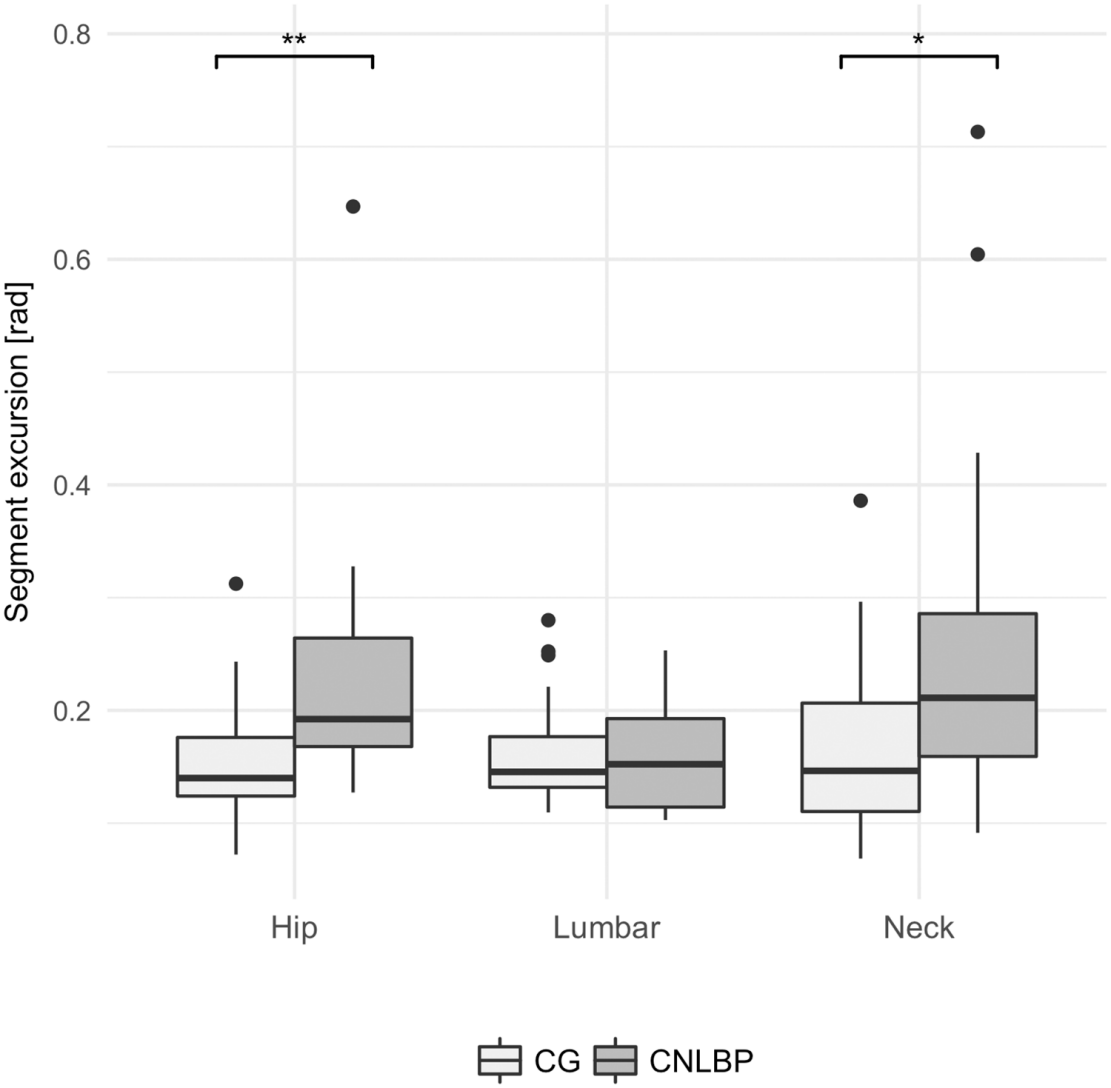
Group comparison of segmental angle excursions. Distribution of the angle excursions of the investigated segments with highest PCA loading. Across trial average values computed for the first second of the active response phase. *P < .025; **P < .001.

The analysis of principal components revealed that the first components, on average, accounted for 89.9% of the variance in CG and 91.5% in the CNLBP group (see Table 2). The first principal component for the CNLBP group accounted for 70.7% of the variance during the response phase. The first principal component for the CG was responsible for 65.5% of the variance. Analyses of the segmental PCA loadings suggest that in the CNLBP group, the neck segment was the principal joint to change the angular position following perturbation while in the CG no single segment had a significantly different impact than the others with a more synergistic distribution across segments. Comparing the rank of variances between the groups revealed that the CNLBP group relied primarily on neck and hip variance to control posture while the CG also involved lumbar flexibility to counter the perturbation. These findings were exploited to reduce the dimensionality of the system and allowed between-group comparison of measured segmental excursion during the response phase in the three segments with the highest loading. This revealed that mean hip angle excursion of patients with CNLBP (*Mdn* = .21*rad*) differed significantly from the pain-free CG (*Mdn* = .15*rad*) during the active response phase (see Fig 6), *W* = 179, *z* = −3.89, *P* < .001, *r* = .51. The first principal component responsible for overall variance, the neck angle excursion, was significantly larger in patients with CNLBP (*Mdn* = .22) than in CG (*Mdn* = .16), *W* = 260, *z* = −2.59, *P* < .025, *r* = .36. Both groups (CNLBP *Mdn* = .16, control *Mdn* = .16) had a similar amount of joint angle excursions on the lumbar segment, *W* = 453, *z* = −.3, *P* = *ns*, *r* = .04.

**Table 2:** Principal component analysis of the involved segments.

Segment	PC1 CG	PC1 CNLBP	PC2 CG	PC2 CNLBP
Foot	.09	.09	.21	.20
Ankle	.23	.18	.26	.31
Knee	.33	.28	.29	.27
Hip	.43	.47	.34	.44
Lumbar	.44	.34	.37	.30
Neck	.46	**.56**	**.52**	**.53**
% of variance	65.50	70.74	24.36	20.74

PC: Principal component; CG: Control group; CNLBP: Chronic non-specific low back pain group. Significant loading (≥ .5) is shown in bold.

### Clinical outcome correlates

Higher pain rating on the VAS scale was correlated with lower relative variance (*rho* = −.46;*P* < .05) and patients with higher pain levels had less predictable CP time-series during the active response phase, shown with a moderate correlation of ApEn with the VAS (*rho* = −.42, *P* < .05) and a non-significant correlation with the ODI-D (*rho* = −.36, *P* = .*ns*). No correlations were observed in CEA (*rho* = −.05 and *rho* = −.002, *ns*) with the ODI and the VAS, respectively.

## Discussion

The presented study shows how differences in postural strategies of a representative sample of CNLBP patients differ on segmental level when compared to symptom-free participants. Both groups were able to regain steady-state joint configuration. This was reflected by higher variance within the uncontrolled manifold compared to the orthogonal sub-space and the resulting relative ratio greater than zero and is consistent with previous findings describing control of undesirable deviation of task goals rather than control of each segment to reach that goal [31, 32, 41]. However, patients with CNLBP seemed to invest more nonmotor equivalent segmental variance to achieve this result. Although not statistically significant, the medium-sized effect underlines the additional effort observed in the CNLBP group to maintain a desirable joint configuration. Significantly more hip- and neck-segment movement were observed in the CNLBP group, whereas neither structure (ApEn) or magnitude (CEA) of the CP trajectory suggest any differences of postural sway. This contradicts findings of a systematic literature review on CP excursion in patients with CNLBP [8]. In their review, Ruhe et al. conclude that CNLBP does affect postural control, particularly under visual deprivation or platform perturbation [8]. However, as has been highlighted by a more recent systematic review on the topic, differences between groups strongly depend on the experimental condition [7]. Ruhe et al. [8] do not strictly differentiate between experimental conditions, but summarise all findings causing considerate heterogeneity regarding the assessed postural tasks of the compared studies [7]. When comparing similar studies with similar experimental conditions, the findings are highly inconsistent [7]: Across all experimental conditions, some studies suggest there is a difference while a similar number of studies suggest there is none. Moreover, the overall quality of the studies with positive findings has been reported to be lower than the studies findings no differences [7].

Nevertheless, possible factors that may have masked the effect of CNLBP on postural sway should be discussed. In this respect, it should be highlighted that the included population had low to moderate pain levels and low disability scores. Although there seems to be no association between pain intensity and the magnitude of postural sway, studies reporting such differences often included patients with higher levels of pain [7]. Moreover, while the pain levels were low on the day of measurement, most patients in this study reported having had severe pain in the past, often limiting daily activities to an immobilising extent. Such experiences have been associated with postural anxiety due to fear of recurring pain [45] and studies on preparatory postural adjustment have shown that prior to perturbation, young patients with recurring pain adopt a slight anterior inclination to increase stability [46]. While we have not analysed such an anticipatory adjustment in this study, it is a possible strategy to achieve better postural results and should be considered for the instruction of the postural tasks [7].

As mentioned earlier, nonlinear measures (e.g. ApEn) are thought to be more sensitive than linear measures (e.g. CEA) to detect subtle differences between time-series data from different groups [35]. Low values of ApEn represent reduced variability related to more rigid and unphysiological movement patterns [47]. This has been demonstrated in patients with cerebral concussions, who had significantly decreased ApEn values for simple postural tasks [47]. It has also been shown that patients with higher levels of pain (numeric pain rating scale ≥ 4) have significantly lower sample entropy, but the same magnitude, when compared to a group of patients with low levels of pain during quiet standing with eyes closed [48]. While our secondary findings presented here also show moderate correlation of pain intensity with nonlinear measures, but not with CEA, the primary findings suggest there are no differences in signal entropy in patients with no pain compared to the CNLBP group. As opposed to Sipko et al. [48], who used sample entropy, we used ApEn to measure the predictability within the CP time-series. Although ApEn has previously been recommended for CP measures of posture [19], a more recent experimental study suggests sample entropy may be more reliable, particularly for shorter data sets [49]. Whether this applies to CP-derived data should be analysed in a future study, although our exploratory comparison of both approaches did not reveal any differences. Another likely reason for these contradicting findings compared to the study by Sipko et al. [48] may relate to the experimental condition of the postural task. It has been shown, and this is in line with the findings by Sipko et al. [48], that increased postural challenge is associated with higher cognitive demands and lower entropy [50]. Standing on a hard surface is posturally less challenging than standing on an oscillating platform after external perturbation. This may explain why the ApEn values were equally low for both groups, as the destabilised platform is challenging for healthy as well as pain-affected people.

The observed variance within motor equivalence similar in both groups contrasts the findings of Tajali et al. [31], who found significantly lower values in an LBP group during a sit-to-stand task. The group concluded that LBP patients adopted a more rigid strategy during the dynamic phase of the task. These discrepancies may be explained by changes in movement patterns when pain persists for years, as the study population of the presented study were older than in Tajali et al. [31] and, on average, had been suffering from pain for decades. The lower motor equivalent variance in young patients with low levels of CNLBP observed in Tajali et al. [31], can be explained with well-described protective compensation methods in early stages of pain occurrence (e.g. rigid muscle activity with low flexibility) [13, 51]. Segmental movement patterns revealed by PCA in a study by Wang et al. [27] suggest a shift from lumbar segmental control to lower limb activity in patients from pre- to post spinal surgery. While these constraints on susceptible areas may be advantageous in the short term, sustained pain for years may have quite the opposite effect [13]. Based on our observations, a long-term follow-up hypothesis could investigate whether lingering and persistent moderate pain may lead to increased variance, indicative of new postural control strategies adopted to cope with dynamic environments. Higher flexibility and complexity may lead to excessive motion outside the physiological limits of spine-stabilizing passive structures [9], thereby contributing to pain sustenance. This interpretation would coincide with the observed larger angle excursion and their disproportionate loading revealed by the PCA as well as the higher nonmotor equivalent variance in the CNLBP group.

### Limitations

It has been suggested that postural control should only be analysed under sufficiently perturbed, dynamic circumstances [8, 51]. In this sense, it might be argued that for the present study, the perturbation caused by the swaying platform while standing on both legs was insufficient to provoke abnormal responses. The limited deflection was chosen as a conservative approach in order to prevent participants from stepping too soon or raising the heel to counter the perturbation and maximise standardisation.

No age-matched screening was planned as the effect of age has been reported to be low [52] and only significant in populations older than 70 [41, 52]. Because of the significant age difference between the CNLBP group and CG, the analysis was repeated without age-specific outliers, but no difference in the findings was found. Future studies should, nevertheless, aim to achieve parallel comparisons to control for this potential confounder. Further caution is advised when comparing the presented results with similar perturbation studies. In the sit-to-stand task and for the perturbation tasks, the beginning and end of the dynamic phases are clearly defined. In the postural sway task, the participants remain on a labile platform throughout the measurement which means the instability is given throughout the task and only rarely would the exact pre-perturbation configuration be regained. It is therefore difficult to isolate the intrinsic variability from the mechanical effects caused by the swaying platform. It has been shown repeatedly, however, that coordination of joint angles primarily originates from active coordination among the elemental variables [23, 28].

A limitation in marker tracking is the inherent discrepancy from actual joint angles and anatomical reference positions caused by soft tissue deformability and marker positioning accuracy [38, 39]. Using only 2D analysis in the sagittal plane has also been reported to increase the possibilities of errors [39]. However, in cases of movement limited predominantly to one plane results are comparable to 3D analysis [39].

This study presents an analysis of the described parameters measured at one point in time. No causal claims are made with regard to the results. Long-term longitudinal studies would allow implications on how motor equivalence and individual joint contribution may change over time and with pain development. The effect of a postural specific intervention on both UCM variance and joint angle excursion would allow description of the direct link between pain, the applied intervention and postural control. Other factors should also be considered, such as fear of falling, exact activity levels, or segmental proprioception.

## Conclusion

In conclusion, this study supports the notion that summary outcomes do not suffice to identify postural deficiencies in CNLBP patients and should be applied in combination with multi-segmental analysis. Significant higher angle variations of the hip segment were needed by patients with CNLBP to maintain similar stability as the symptom-free CG. Yet, CP outcomes and the proposed UI model did not reflect such differences, suggesting limited clinical use of the measure in patients with CNLBP. When assessing postural control on labile platforms in patients with moderate CNLBP, clinicians using kinematic assessments should observe individual segments with particular attention on excessive hip and neck motion.
